# Knockdown of Claudin-8 (CLDN8) Indicates a Link Between Breast Cancer Cell Sensitivity to Chemotherapeutics and Reveals a Potential Use of CLDN8 as a Molecular Diagnostic and Target for Therapy

**DOI:** 10.3390/ijms26115412

**Published:** 2025-06-05

**Authors:** Wenxiao Ji, Yufei Lou, Wen G. Jiang, Fiona Ruge, Tracey A. Martin

**Affiliations:** Cardiff China Medical Research Collaborative (Ccmrc), School of Medicine, Cardiff University, Heath Park, Cardiff CF14 4XN, UK; jiw3@cardiff.ac.uk (W.J.); louy6@cardiff.ac.uk (Y.L.); jiangw@cardiff.ac.uk (W.G.J.); ruge@cardiff.ac.uk (F.R.)

**Keywords:** Claudin-8, breast cancer, predictive biomarker, therapeutic target, drug sensitivity, treatment response

## Abstract

Breast cancer is a heterogeneous disease, and treatment resistance remains a critical challenge. Claudin-8 (CLDN8), a tight junction protein, has emerged as a potential indicator of therapeutic response and prognosis in breast cancer patients. In this study, we evaluated CLDN8 as a predictive biomarker and a potential therapeutic target. We analyzed CLDN8 gene expression in breast cancer patient cohorts to assess its association with clinical outcomes and response to therapy. We also established breast cancer cell models with altered CLDN8 expression to examine its effects on cell behavior and drug sensitivity. High CLDN8 expression was significantly associated with improved disease-free survival, particularly in estrogen receptor-negative patients (*p* = 0.007), suggesting a favorable prognostic role. Notably, tumors with elevated CLDN8 showed better outcomes in patients treated with surgery alone or endocrine therapy, whereas in those receiving chemotherapy (including neoadjuvant) or anti-HER2 therapy, high CLDN8 levels were paradoxically linked to poorer survival and therapy resistance. In vitro, CLDN8 knockdown reduced sensitivity to endocrine treatments, HER2-targeted agents, and chemotherapeutic drugs, mirroring clinical patterns. In conclusion, our findings identify CLDN8 as an important prognostic factor in breast cancer and as a novel predictor of treatment response. These results underscore the potential utility of CLDN8 status in guiding personalized therapy and highlight CLDN8 as a candidate target for overcoming treatment resistance in breast cancer.

## 1. Introduction

Breast cancer poses a significant global health challenge, with its complexity stemming from diverse molecular subtypes that exhibit distinct responses to various treatment modalities [1]. Among the five different subtypes, two prominent challenges have emerged in the management of breast cancer: resistance to endocrine therapy in estrogen receptor ER(+) tumors [2] and heterogeneous response to anti-HER2 therapy in HER2(+) tumors [3]. In ER(+) breast cancer, resistance to endocrine therapy often develops, limiting the effectiveness of treatment [4]. Additionally, patients with HER2(+) breast cancer may exhibit varying responses to anti-HER2 targeted therapies, with some developing resistance over time [5]. These challenges highlight the urgent need for novel predictive biomarkers and therapeutic targets to guide treatment decisions and improve outcomes in patients with breast cancer.

Loss of cell–cell adhesion is a hallmark of cancer progression and metastasis, allowing tumor cells to disseminate from the primary site [6]. Tight junction proteins, notably the claudin (CLDN) family, are critical for maintaining epithelial cell adhesion, polarity, and barrier function [7]. Aberrant expression of claudins can compromise junctional integrity and has been implicated in tumor invasion and metastasis in various cancers, including breast cancer [8]. Indeed, several claudin family members have been identified as prognostic indicators or mediators of breast cancer progression. For example, reduced CLDN1 expression correlates with higher recurrence rates and poorer survival in breast cancer [9,10]. Conversely, CLDN2 overexpression has been shown to promote breast cancer metastasis to the liver through interactions between tumor cells and hepatocytes [11,12]. Alterations in other claudins further underscore their diverse roles: elevated CLDN4 expression is associated with lymph node metastasis and enhanced cancer cell stemness, while loss of CLDN6 can increase breast cancer cell motility and resistance to apoptosis, facilitating invasion [13,14,15,16].Another study suggested that CLDN6 is transcriptionally upregulated by HIF-1α under hypoxic conditions [17]. Loss of CLDN6 may lead to increased HIF-1α-driven breast cancer metastasis in a SUMOylation-dependent manner [18]. Martin et al. [19] demonstrated that overexpression of CLDN20 in breast cancer cells decreases TER and thus increases their motility and reduced trans-epithelial resistance. Collectively, these findings suggest that the dysregulation of tight junction molecules is an important contributor to breast cancer aggressiveness and that specific claudins may serve as valuable prognostic biomarkers.

Among the Claudin family, CLDN8 remains relatively unexplored in breast cancer. CLDN8 is known to participate in tight junction formation and epithelial cohesion; however, its function in tumor biology is not well defined [8]. Early evidence indicates that CLDN8 expression is frequently downregulated in breast tumors [20]. Clinically, low CLDN8 levels have been associated with a higher incidence of lymph node metastasis and poorer outcomes in patients with breast cancer [20]. Notably, CLDN8 expression appears to positively correlate with androgen receptor (AR) status in breast tumors, and patients with concomitantly low CLDN8 and low AR expression have particularly unfavorable prognoses [20]. These observations suggest that CLDN8 may act as a context-dependent tumor suppressor or marker of a less aggressive, more differentiated tumor phenotype. However, the role of CLDN8 in influencing the response to therapy remains unclear. Given the persistent challenges of endocrine resistance in ER(+) disease and treatment failure in some HER2(+) cases, understanding whether CLDN8 impacts sensitivity to these therapies could be key to improving treatment strategies. In this study, we set out to investigate the significance of CLDN8 in breast cancer outcomes and treatment response. We hypothesized that CLDN8 expression may stratify patient prognosis and predict their responsiveness to different therapies. To test this hypothesis, we analyzed clinical breast cancer specimens to correlate CLDN8 expression with disease-free survival and treatment efficacy across various patient subgroups. We further performed in vitro experiments using breast cancer cell lines with modulated CLDN8 expression (knockdown and control) to assess the influence of CLDN8 on cellular behavior and drug sensitivity. A panel of nine therapeutic agents was selected to reflect the major treatment modalities in breast cancer care: chemotherapeutic drugs (Docetaxel, Paclitaxel, Cisplatin, and Methotrexate), HER2-targeted tyrosine kinase inhibitors (Lapatinib and Neratinib), and endocrine therapies (Tamoxifen, Fulvestrant, and Anastrozole). By examining CLDN8’s impact on sensitivity to these treatments, we aimed to bridge our laboratory findings with clinical relevance. Although our mechanistic studies were conducted in vitro, the chosen drugs mirror standard clinical therapies, allowing us to infer how alterations in CLDN8 might affect patient responses in real-world settings. Through this comprehensive approach, we address whether CLDN8 can serve as a predictive biomarker for therapy outcomes and explore the potential of targeting CLDN8 in future breast cancer treatment strategies. Our work thereby seeks to clarify the clinical value of CLDN8 in breast cancer and lay a foundation for more personalized therapeutic interventions.

## 2. Results

### 2.1. Expression of CLDN8 in Breast Cancer

**Figure 1:**
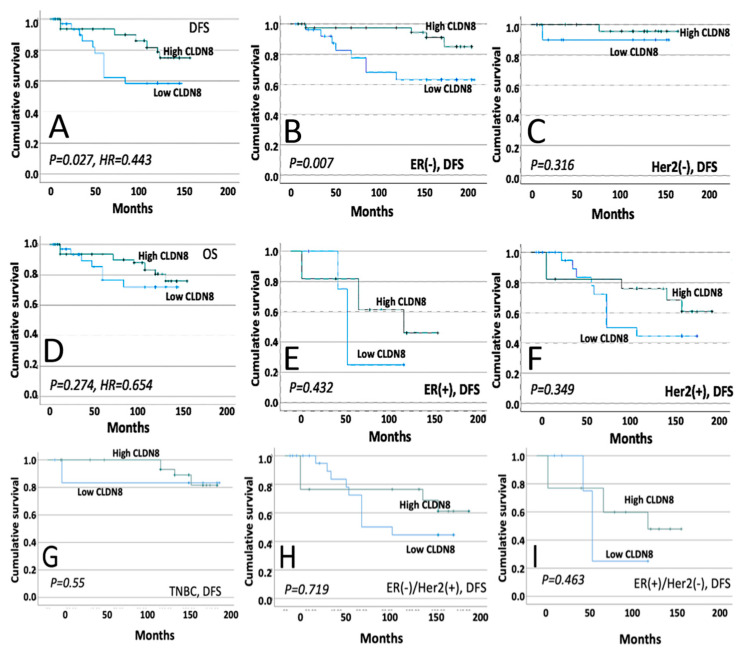
DFS in high vs. low CLDN8 levels (**A**); ER(−) patient DFS with CLDN8 levels (**B**); HER2(−) patient DFS with CLDN8 levels (**C**); OS in high vs. low CLDN8 levels (**D**); ER(+) patient DFS with CLDN8 levels (**E**); HER2(+) patient DFS with CLDN8 levels (**F**); TNBC patient DFS with CLDN8 levels (**G**); ER(−)/HER2(+) patient DFS with CLDN8 levels (**H**); ER(+)/HER2(−) patient DFS with CLDN8 levels (**I**). “DFS” stands for disease-free survival, “OS” stands for overall survival, “ER” refers to estrogen receptor status, “HER2” refers to human epidermal growth factor Receptor 2 status, and “TNBC” stands for triple-negative breast cancer.

Data from the Cardiff cohort were used to investigate the prognostic significance of CLDN8 expression in breast cancer. As shown in Figure 1, a total of 102 cases were included in the analysis. CLDN8 expression levels were categorized into high and low groups based on the median expression values within the cohort. Samples with expression levels above the median were classified as the high-expression group (*n* = 67), while those below the median were classified as the low-expression group (*n* = 35).

Higher expression levels of CLDN8 were significantly correlated with improved disease-free survival (DFS), as indicated by a hazard ratio of 0.443 and a *p*-value of 0.027 (Figure 1A). This association was notably pronounced in estrogen receptor-negative (ER(−)) patients, where high CLDN8 expression was markedly linked to better DFS outcomes (*p* = 0.007) (Figure 1B). Conversely, in the context of human epidermal growth factor receptor 2 (HER2) status, CLDN8 expression did not demonstrate a significant prognostic value for DFS among HER2(−) patients (*p* = 0.316) (Figure 1C), suggesting that its predictive capacity may not extend to this subgroup. Furthermore, our analysis did not reveal a significant association between CLDN8 levels and overall survival (OS) in the studied cohort (*p* = 0.274) (Figure 1D), nor did it substantiate the role of CLDN8 as a determinant of DFS in either ER(+) (*p* = 0.432) (Figure 1E) or HER2(+) (*p* = 0.349) (Figure 1F)breast cancer patients. These patterns persisted upon further stratification, with CLDN8 expression not serving as a significant predictor of DFS in patients with triple-negative breast cancer (*p* = 0.55) (Figure 1G), ER(−)/HER2(+) (*p* = 0.719) (Figure 1H), or ER(+)/HER2(−) (*p* = 0.463) (Figure 1I). Collectively, these findings underscore the potential of CLDN8 as a marker of favorable prognosis in ER(−) breast cancer, while also highlighting the complexity of its role across various breast cancer subtypes, thus necessitating additional research to elucidate its clinical utility.

### 2.2. Association of CLDN8 Expression with Clinicopathological Factors

**Figure 2:**
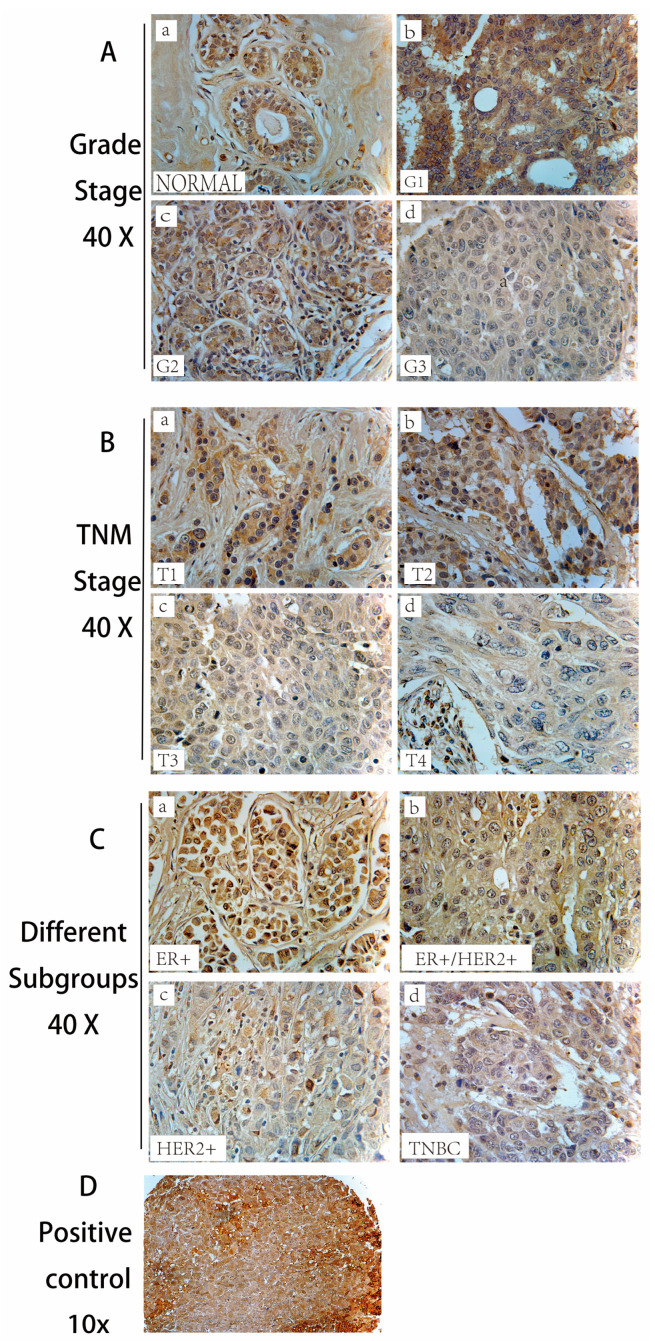
Immunohistochemical (IHC) staining of CLDN8 expression in breast cancer tissue. (**A**) CLDN8 expression in normal breast tissue and breast cancer of different histological grades. (**a**) Normal breast tissue, (**b**) Grade 1 (G1), (**c**) Grade 2 (G2), (**d**) Grade 3 (G3). (**B**) CLDN8 expression in different TNM stages of breast cancer. (**a**) T1, (**b**) T2, (**c**) T3, (**d**) T4. (**C**) CLDN8 expression in different breast cancer subtypes. (**a**) ER(+), (**b**) ER(+)/HER2(+), (**c**) HER2(+), (**d**) TNBC (triple-negative breast cancer). (**D**) Positive control image at 10× magnification.

IHC analysis revealed distinct alterations in CLDN8 expression across different histological grades of breast cancer (Figure 2Aa). In normal breast tissue, CLDN8 was primarily localized to the cell membrane of ductal epithelial cells and showed mild cytoplasmic staining. In Grade 1 tumors (Figure 2Ab), CLDN8 expression was enhanced and predominantly membrane-localized, but with increased cytoplasmic staining, indicating partial disruption of normal tight junction functionality. As the tumor grade progressed, the CLDN8 expression became more diffuse. In Grade 2 tumors (Figure 2Ac), there was a noticeable shift toward cytoplasmic staining with reduced membrane localization, suggesting a loss of cell−cell adhesion integrity. In Grade 3 tumors (Figure 2Ad), CLDN8 expression was significantly reduced, exhibiting a homogeneous staining pattern with minimal membrane localization, further indicating the loss of barrier function in high-grade breast cancer. These findings suggest that CLDN8 undergoes a marked transition from membrane localization in normal ducts to diffuse cytoplasmic expression in high-grade tumors, with a substantial reduction in Grade 3 cancers.

To further investigate the role of CLDN8 in tumor progression, IHC staining was performed across different TNM stages (Figure 2B). In early stage tumors (T1) (Figure 2Ba), CLDN8 was primarily localized to the cell membrane with observable cytoplasmic staining, maintaining high expression levels and indicating partial preservation of tight junction function. As the tumor advanced to T2 (Figure 2Bb), membrane localization weakened, and cytoplasmic staining became more prominent, suggesting a decline in cell−cell junction integrity. In T3 tumors (Figure 2Bc), CLDN8 expression intensity decreased further, with heterogeneous staining patterns and a shift toward cytoplasmic expression, indicating progressive functional loss. By T4 (Figure 2Bd), CLDN8 expression was significantly diminished, with almost no membrane localization and only faint cytoplasmic staining in a few cells. These results suggest that as tumors progress to higher TNM stages, CLDN8 expression declines, potentially contributing to increased invasiveness and tumor aggressiveness.

CLDN8 expression was also assessed in different molecular subtypes of breast cancer, including ER(+), HER2(+), ER(+)/HER2(+), and triple-negative breast cancer (TNBC) subtypes (Figure 2C). In ER(+) breast cancer (Figure 2Ca), CLDN8 was primarily membrane-localized with moderate cytoplasmic staining, indicating retained tight junction function. In ER(+)/HER2(+) tumors (Figure 2Cb), membrane localization was preserved, but cytoplasmic staining increased, suggesting a potential influence of HER2-related signaling on CLDN8 distribution. In HER2(+) tumors (Figure 2Cd), CLDN8 expression was reduced, exhibiting a more diffuse staining pattern with diminished membrane localization, implying that HER2 signaling may promote CLDN8 downregulation or functional alteration. TNBC (Figure 2Cd) showed the lowest CLDN8 expression levels, with almost no membrane staining and weak or absent cytoplasmic staining, indicating further loss of function. The stark contrast in CLDN8 expression across these subtypes suggests a potential role in tumor biology, particularly in cell adhesion, invasion properties, and response to therapy.

Quantitative analysis of CLDN8 staining intensity across different breast cancer subtypes, grades, and TNM stages further confirmed these findings (Table 1). CLDN8 expression varied significantly across tumor grades, with a marked reduction in Grade 3 compared to Grade 1 (*p* = 0.022). Similarly, CLDN8 expression decreased significantly at higher TNM stages, with T3 and T4 tumors showing the lowest expression levels (*p* < 0.0001). Among the molecular subtypes, HER2(+) tumors exhibited a significant reduction in CLDN8 expression compared to normal tissue (*p* = 0.03), while TNBC demonstrated the most dramatic loss of CLDN8 expression (*p* = 0.01). These findings suggest that CLDN8 downregulation may contribute to the aggressive behavior of high-grade and late-stage tumors, particularly in TNBC.

### 2.3. CLDN8 Expression in Breast Cancer Treatment Modalities

**Figure 3:**
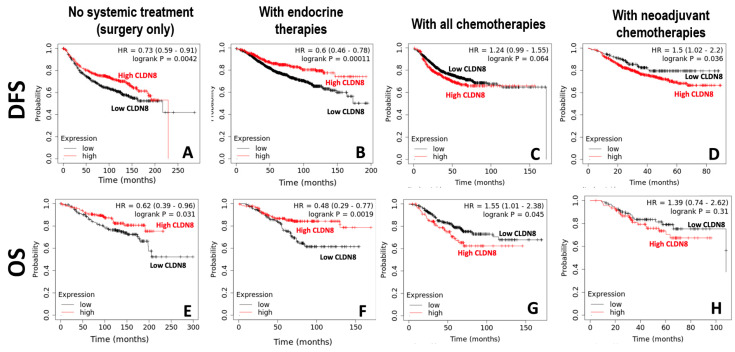
DFS with surgery only: CLDN8 expression impact (**A**); DFS with endocrine therapy: CLDN8 expression levels (**B**); DFS with all chemotherapies: CLDN8 expression contrast (**C**); DFS with neoadjuvant chemotherapies: CLDN8 high vs. low (**D**); OS with surgery only: high vs. low CLDN8 expression (**E**); OS with endocrine therapy: impact of CLDN8 expression (**F**); OS with all chemotherapies: comparing CLDN8 levels (**G**); OS with neoadjuvant chemotherapies (**H**).

The patients were divided into high and low CLDN8 expression groups using a cut-off value obtained from the ROC analysis. Figure 3A presents a scenario without systemic treatment, where a higher expression of CLDN8 is associated with improved disease-free survival (DFS), as suggested by a hazard ratio (HR) of 0.73 (*p* = 0.0042). Similarly, in patients who received endocrine therapy, high levels of CLDN8 expression also appeared to be a favorable indicator (HR = 0.63, *p* = 0.00011) (Figure 3B). In contrast, for patients who received neoadjuvant chemotherapy, high levels of CLDN8 were an indicator of poor clinical outcome (HR = 1.5, *p* = 0.026) (Figure 3D). A similar pattern was observed in those who received comprehensive chemotherapy treatments, although this was not statistically significant (HR = 1.24, *p* = 0.064) (Figure 3C).

This connection was similarly observed when assessed by overall survival (OS) (Figure 3E–H), in that high CLDN8 expression was a good prognostic indicator in those who underwent surgery only (Figure 3E) and endocrine therapies (Figure 3F), but an indicator for poor prognosis for those who received systemic chemotherapies (Figure 3G) and to a limited degree, neoadjuvant chemotherapies (Figure 3H). These data validate the foundational theory that high CLDN8 expression signifies endocrine sensitivity but may also indicate resistance to chemotherapy, especially in a pre-surgical setting, potentially serving as a pivotal factor in tailoring personalized treatment strategies and enhancing therapeutic efficacy for patients with breast cancer.

**Figure 4:**
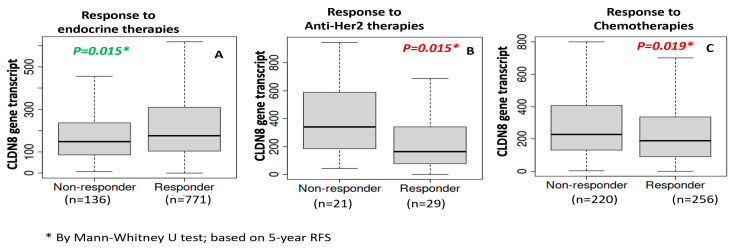
CLDN8 expression in endocrine therapy response (**A**); CLDN8 levels in anti-HER2 therapy response (**B**); CLDN8 expression and chemotherapy response (**C**).

We further explored databases in which levels of CLDN8 were compared between patients who were sensitive and resistant to different treatments (www.rocplot.org). High CLDN8 expression was associated with increased sensitivity to endocrine therapies, as evidenced by higher levels in responders (*p* = 0.015) (Figure 4, left). Conversely, elevated CLDN8 levels correlated with resistance to Anti-HER2 and chemotherapy treatments, where non-responders exhibited higher expression (*p* = 0.015 and *p* = 0.019, respectively) (Figure 4, middle and right). Therefore, patients with lower CLDN8 expression may be more responsive to these treatments, indicating the potential of CLDN8 as a differential biomarker for tailoring breast cancer therapy.

### 2.4. IC50 Values of the Cytotoxicity Assays in the Breast Cancer Cell Model

**Figure 5:**
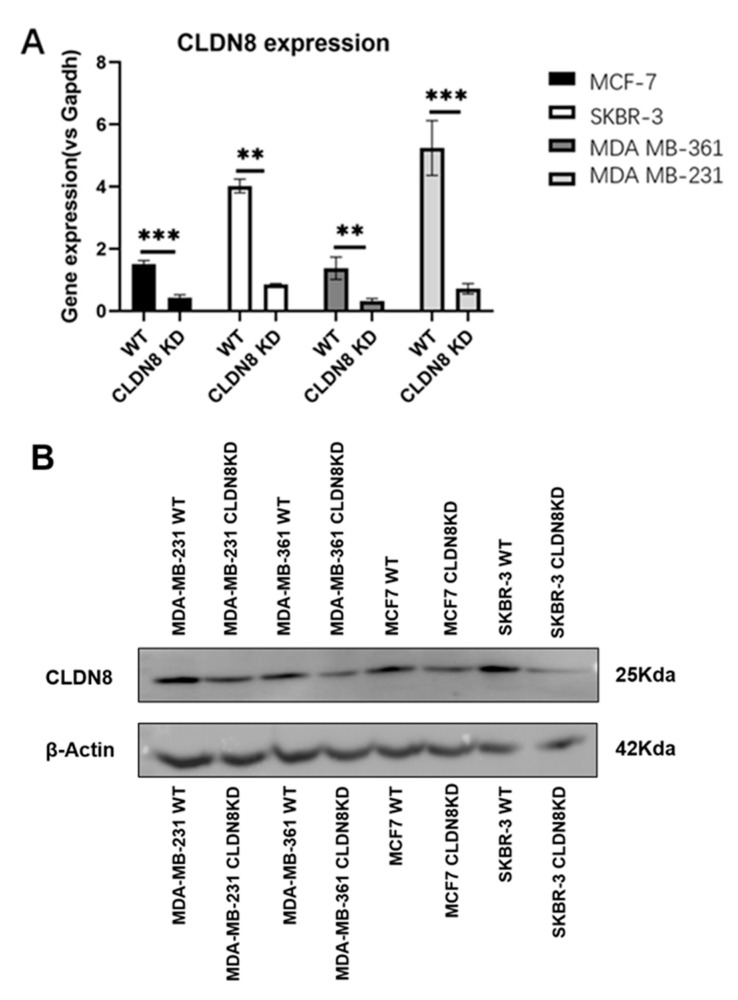
Expression of CLDN8 in breast cancer cell lines. (**A**) qPCR analysis confirming the knockdown efficiency in MDA-MB-231, MDA-MB-361, SKBR3, and MCF-7 cells. Fold changes were calculated using the 2−∆∆Ct method; statistical significance was determined using an unpaired t-test (** *p* < 0.01, *** *p* < 0.001). (**B**) Western blot of CLDN8 protein levels in WT and KD cells; β-actin was used as a loading control. WT, wild type; KD, knockdown.

The knockdown efficiency of CLDN8 across breast cancer cell lines was confirmed by qPCR and Western blot (Figure 5). To further delineate the impact of CLDN8 on drug sensitivity, we compared the IC50 values between CLDN8 wild-type (WT) and CLDN8 knockdown (KD) breast cancer cell lines for a panel of therapies (Table 2). A consistent pattern emerged across all four representative cell models (ER^+^/HER2^−^ MCF-7, HER2^+^/ER^−^ SKBR-3, HER2^+^/ER^+^ MDA-MB-361, and triple-negative MDA-MB-231), a consistent pattern emerged. CLDN8 knockdown cells were less sensitive to endocrine therapy but more sensitive to anti-HER2 and chemotherapeutic agents than WT cells. For example, the IC50 for Tamoxifen (an ER antagonist) was markedly higher in CLDN8KD cells than in WT (indicating that a greater drug concentration was required to inhibit growth in the absence of CLDN8). A similar upward shift in IC50 was observed for other hormonal treatments like Fulvestrant and Anastrozole in CLDN8KD cells, reflecting a relative resistance to endocrine therapy when CLDN8 expression is lost. In contrast, CLDN8KD cells exhibited significantly lower IC50 values for Lapatinib (*p* < 0.05), Neratinib (*p* < 0.01), and taxanes (Docetaxel and Paclitaxel, *p* < 0.01). However, the differences between Cisplatin and Methotrexate were not statistically significant (*p* > 0.05). This downward shift in the IC50 upon CLDN8 silencing indicates that the loss of CLDN8 consistently enhances sensitivity to cytotoxic and HER2-targeted agents. Notably, statistically significant differences in IC50 values were observed for endocrine therapies (e.g., Tamoxifen, *p* < 0.01) and HER2-targeted agents (e.g., Lapatinib, *p* < 0.05), whereas differences for chemotherapeutics such as Cisplatin and Methotrexate did not reach statistical significance (*p* > 0.05). These results suggest that CLDN8 expression selectively modulates the therapeutic response to hormone-based and HER2-targeted treatments in vitro. Taken together, the IC50 analysis suggests a dual role for CLDN8: promoting responsiveness to hormone-based treatments (since its absence induces resistance) while conferring a measure of protection against HER2 inhibitors and chemotoxic drugs (since its absence increases sensitivity).

### 2.5. CLDN8 Expression and Endocrine Therapy Response in Different Subgroups

**Figure 6:**
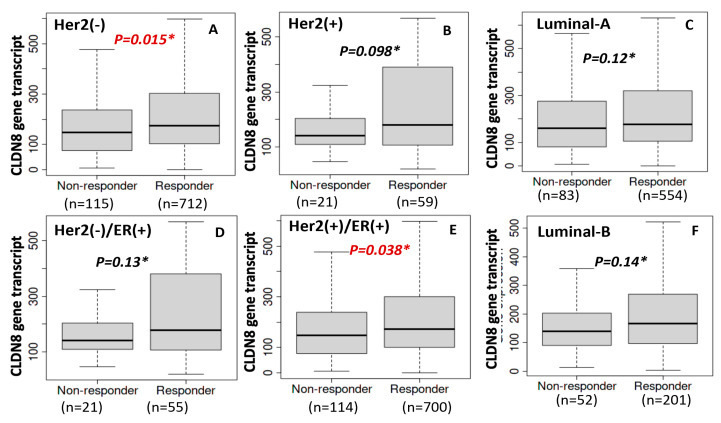
CLDN8 and endocrine therapy response with ER and HER2, and 5-year RFS. CLDN8 Expression in HER2(−) Breast cancer patients (**A**); CLDN8 levels in HER2(+) patients (**B**); Differential CLDN8 expression in luminal-A subtype (**C**); CLDN8 expression in HER2(−)/ER (+) Breast cancer (**D**); HER2(+)/ER (+) Breast cancer (**E**); luminal-B breast cancer (**F**).

Building on the IC50 findings, we examined the relationship between CLDN8 and endocrine therapy outcomes, both clinically and in cell-based growth assays. Figure 6 focuses on patients who received adjuvant endocrine therapy, stratified by molecular subgroups, and highlights a clear association between high CLDN8 expression and a favorable treatment response. In these cohorts, patients were divided into CLDN8-high and CLDN8-low groups (using an optimal ROC-derived cut-off) and evaluated for 5-year relapse-free survival (RFS). Consistently, patients with higher CLDN8 levels had better 5-year RFS under endocrine therapy than those with low CLDN8 levels. This trend was particularly evident in the ER^+^/HER2^−^ subgroup (luminal A phenotype; Figure 6A,D), where non-relapsing patients showed significantly higher CLDN8 expression than those who experienced recurrence within 5 years. A similar positive association, although somewhat attenuated, was observed in the ER^+^/HER2^+^ subgroup (luminal B; Figure 6B,E) and in all HER2(−) endocrine-treated patients. In these groups, CLDN8-high tumors were more likely to respond to hormonal therapy, consistent with improved RFS. Notably, when considering intrinsic subtypes, as shown in Figure 6, luminal A patients (typically low-grade ER^+^ tumors) had the most pronounced CLDN8-related benefit, whereas the luminal B subgroup (often more proliferative or HER2^+^ ER^+^ tumors; Figure 6F) showed a positive trend that did not reach strong significance. These clinical data reinforce that CLDN8 expression serves as a favorable biomarker of endocrine therapy efficacy across diverse patient subsets, presumably because CLDN8-rich tumors maintain an epithelial, hormone-dependent state that responds well to ER-targeted treatments.

**Figure 7:**
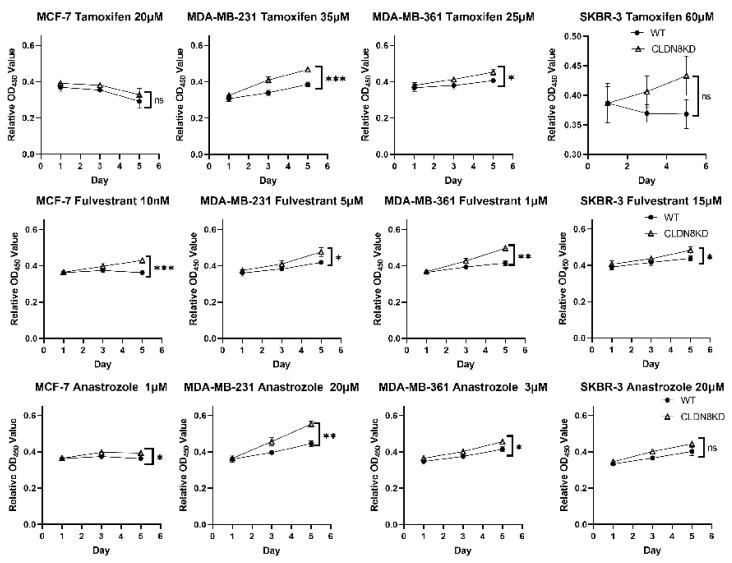
CLDN8 knockdown attenuates the inhibitory effects of endocrine therapy on breast cancer cell proliferation. Growth assays were performed on CLDN8 wild-type (WT) and knockdown (KD) cell lines (MCF-7, MDA-MB-361, MDA-MB-231, and SKBR-3) treated with Tamoxifen, Fulvestrant, or Anastrozole. Statistical significance: *p* < 0.05 (*), *p* < 0.01 (**), *p* < 0.001 (***), versus WT controls under the same treatment conditions.

Complementing the clinical observations, we first verified the knockdown efficiency of CLDN8 in various breast cancer cell lines (MCF-7, SKBR-3, MDA-MB-361, and MDA-MB-231). Figure 7 demonstrates how CLDN8 functionally influences endocrine therapy response in vitro, as shown by detailed growth assays. Breast cancer cells (MCF-7, MDA-MB-361, MDA-MB-231, and SKBR-3) with or without CLDN8 were treated with endocrine agents, and their viability and proliferation were monitored. We then assessed long-term growth under a fixed sub-lethal dose of Tamoxifen. Over the 5-day treatment period, WT and CLDN8KD cells displayed divergent proliferation outcomes. In estrogen receptor– lines, the presence of CLDN8 was associated with greater growth suppression by Tamoxifen. For instance, in MDA-MB-361 cells (ER^+^/HER2^+^), WT cultures showed a sharp growth deceleration under Tamoxifen, whereas CLDN8KD cells continued to proliferate more rapidly, resulting in a significantly higher cell count by day 5 (*p* < 0.05). Similarly, MDA-MB-231 cells (ER^−^, serving as a control for off-target effects) showed that WT cells were moderately inhibited by a high dose of Tamoxifen, while CLDN8KD cells were even less affected, culminating in a highly significant difference in cell growth by day 5 (***, *p* < 0.001). In contrast, MCF-7 cells (ER^+^/HER2^−^) were strongly growth-inhibited by Tamoxifen and both WT and CLDN8KD showed comparable suppression (no significant difference), suggesting that in this highly endocrine-sensitive cell line, loss of CLDN8 could be partially compensated by robust ER signaling dependence. As expected, SKBR-3 cells (HER2^+^/ER^−^, which lack the ER target) did not respond to Tamoxifen and accordingly showed no growth difference between WT and CLDN8KD under treatment (“ns”).

We observed analogous results with other hormone therapies (data summarized in Figure 7 and Drug IC50 curves supplemental figures). Fulvestrant (an ER degrader) also demonstrated dose-dependent growth inhibition, which was attenuated by CLDN8 knockdown. CLDN8KD cells had slightly higher Fulvestrant IC50 values than WT (Figure 7, Fulvestrant), and in 5-day proliferation assays, they maintained higher growth rates under Fulvestrant treatment. All four cell lines exhibited greater proliferation in CLDN8KD than in WT by day 5 with Fulvestrant, with statistically significant differences emerging, particularly in the ER^+^ models (*p* < 0.01 for MCF-7 and MDA-MB-361; Figure 7, Fulvestrant). Anastrozole (an aromatase inhibitor) was tested in an estrogen-dependent context (with exogenous androgen in the culture for aromatase activity). Consistently, CLDN8KD cells were less inhibited by Anastrozole: their IC50 values increased relative to WT, and CLDN8KD MCF-7, MDA-MB-361, and MDA-MB-231 all showed a significantly higher cell yield by day 5 compared to WT under Anastrozole treatment (*p* < 0.05 or 0.01; Figure 7, Anastrozole). SKBR-3 (ER^−^) cells showed no response to endocrine treatment. These in vitro findings dovetail with patient data; knocking down CLDN8 impairs the efficacy of endocrine treatments, mirroring the poorer outcomes seen in CLDN8-low tumors clinically. Therefore, CLDN8 appears to preserve an endocrine-responsive phenotype, and its high expression correlates with—and functionally contributes to—greater sensitivity to hormonal therapy in breast cancer.

### 2.6. CLDN8 Expression and Anti-HER2 Therapy Response in Different Subgroups

**Figure 8:**
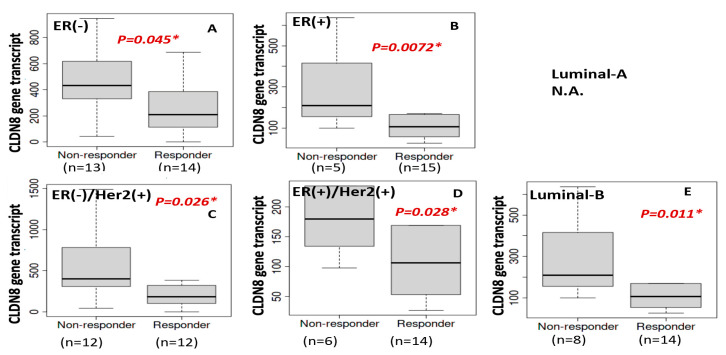
CLDN8 and anti-HER2 therapy response with ER and HER2. CLDN8 expression in ER (−) breast cancer (**A**); in ER (+) patients (**B**); in ER(−)/HER2(+) breast cancer (**C**); in ER (+)/HER2 (+) breast cancer (**D**); and luminal-B breast cancer (**E**).

Next, we explored the role of CLDN8 in HER2-targeted therapies. Given that HER2(+) breast cancers often receive agents such as trastuzumab or kinase inhibitors, we analyzed CLDN8’s association with treatment response in HER2^+^ patient subgroups and validated the findings in HER2-driven cell models. Figure 8 summarizes the clinical data stratified by hormone receptor status within the HER2(+) cohorts. Notably, an inverse relationship between CLDN8 expression and anti-HER2 therapy success was observed. Among HER2(+) patients, those who responded well to anti-HER2 treatment had significantly lower CLDN8 expression than non-responders. This pattern was observed in both major subcategories of HER2^+^ disease. In the ER(−)/HER2(+) group (HER2-enriched subtype), responders exhibited markedly lower CLDN8 levels than patients whose tumors were resistant to HER2 blockade (*p* ≈ 0.01, Figure 8A). Similarly, in ER(+)/HER2(+) (luminal B) patients, non-responders tended to have higher CLDN8 expression, whereas responders had relatively lower expression (Figure 8B,D). The difference in CLDN8 expression between these groups was significant in our dataset (Figure 8D, *p* < 0.05), suggesting that even in ER^+^/HER2^+^ tumors, elevated CLDN8 expression may dampen the benefits of HER2-targeted therapy. When considering intrinsic subtype classification, the luminal B subgroup (which includes many HER2^+^ cases) also showed this inverse trend: patients classified as luminal B who achieved a good response to anti-HER2 therapy had lower CLDN8 expression than those with poor response (Figure 8E, *p* = 0.011). In contrast, in the ER(+) (overall) population without separating HER2 status, CLDN8 differences were less pronounced, indicating that the predictive value of CLDN8 in anti-HER2 settings is most evident when HER2-driven tumors are specifically considered. Together, Figure 8 highlights that high CLDN8 expression is associated with resistance to HER2-targeted treatment, whereas low CLDN8 expression identifies patients who are more likely to benefit from anti-HER2 therapy. This finding complements the endocrine therapy results, highlighting the context-dependent role of CLDN8: beneficial in hormone-driven tumors but potentially detrimental in HER2-driven therapy scenarios.

**Figure 9:**
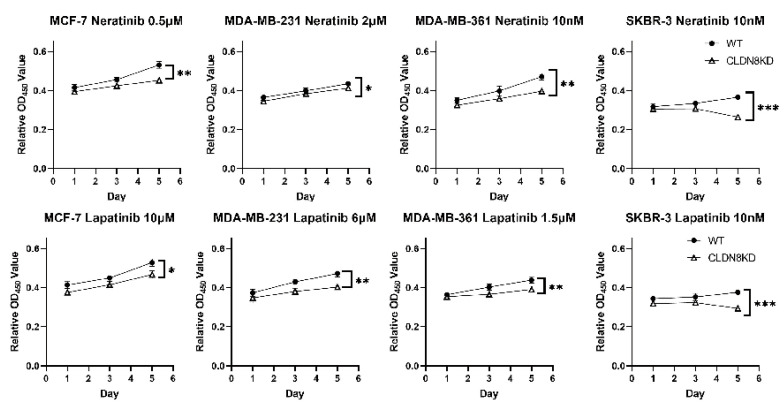
CLDN8 knockdown increases resistance to anti-HER2 therapy in HER2^+^ breast cancer cells. Growth assays were performed on HER2^+^ breast cancer cell lines (SKBR-3 and MDA-MB-361) to compare wild-type (WT) and CLDN8 knockdown (KD) cells treated with Lapatinib or Neratinib. In both cell lines, CLDN8 knockdown significantly enhanced cell proliferation during treatment, indicating a reduced sensitivity to HER2-targeted agents. Statistical significance: *p* < 0.05 (*), *p* < 0.01 (**), *p* < 0.001 (***), versus WT controls under the same treatment conditions.

In vitro experiments corroborated these clinical insights, demonstrating that CLDN8 impairs the efficacy of HER2-targeted drugs at the cellular level. Figure 9 shows the results of Neratinib and Lapatinib growth assays in the four breast cancer cell lines (two HER2^+^ lines: SKBR-3, MDA-MB-361; and two HER2^−^ lines included for comparison: MCF-7 and MDA-MB-231). Across all examined lines, the IC50 for Neratinib in CLDN8KD was slightly lower than that in WT, indicating enhanced drug sensitivity due to CLDN8 loss (Table 2). This trend was even more apparent with Lapatinib (another HER2-directed TKI; dose–response in Figure 9). In every cell line tested, CLDN8 knockdown reduced the IC50 relative to WT, indicating that KD cells required a lower concentration of Lapatinib to achieve 50% growth inhibition compared to WT cells. Next, we assessed cell proliferation over 5 days using a fixed, clinically relevant dose of these drugs (Figure 9). The growth assay results showed that CLDN8 knockdown dramatically improved the growth-inhibitory effect of HER2-targeted therapy. For instance, under Neratinib treatment, CLDN8KD MDA-MB-361, MCF-7, and MDA-MB-231 cultures exhibited significantly lower cell numbers by day 5 compared to their WT counterparts (*p* < 0.05, *p* < 0.01; Figure 9, Neratinib). Notably, in SKBR-3 cells (HER2^+^/ER^−^), which are highly dependent on HER2 signaling, CLDN8 knockdown augmented Neratinib efficacy (although this trend did not reach significance in our assays, the KD curve lay below the WT). Lapatinib exposure yielded concordant outcomes: by day 5, all four CLDN8KD cell lines showed a pronounced reduction in proliferation relative to WT under Lapatinib treatment (*p* < 0.01 in multiple cases; Figure 9, Lapatinib panels). The enhancement was particularly striking in the HER2-driven lines; for example, SKBR-3 cells lacking CLDN8 were far more growth-inhibited (exhibiting ~40% lower cell counts than WT under the same dose by day 5, ** *p* < 0.001), indicating that CLDN8 contributes substantially to Lapatinib resistance. These experiments confirmed that silencing CLDN8 increased cellular responsiveness to HER2 blockade, which was consistent with the patient data in Figure 8. In summary, CLDN8-high status confers relative resistance to anti-HER2 therapies, whereas CLDN8 depletion or low expression sensitizes tumor cells to HER2-targeted growth inhibition.

### 2.7. CLDN8 Expression and Chemotherapy Response in Different Subgroups

**Figure 10:**
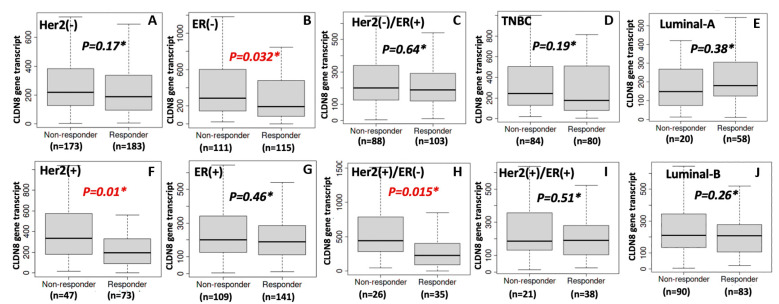
CLDN8 and chemotherapy response with ER and HER2. CLDN8 in HER2(−) response (**A**); in ER(−) response (**B**); in HER2(−)/ER(+) response (**C**); in TNBC response (**D**); in luminal-A response (**E**); in HER2(+) response (**F**); in ER(+) response (**G**); in HER2(+)/ER(−) response (**H**); in HER2(+)/ER(+) response (**I**); in luminal-B response (**J**).

Finally, we investigated the relationship between CLDN8 and the efficacy of standard chemotherapy across breast cancer subtypes. Figure 10 shows the clinical chemotherapy response data stratified by ER and HER2 status, revealing that CLDN8’s predictive impact varies by tumor context. In HER2(−) patients who received chemotherapy (Figure 10A), CLDN8 expression did not differ significantly between responders and non-responders, suggesting that for tumors lacking HER2, high CLDN8 expression is not a strong determinant of chemosensitivity. This category includes both ER^+^/HER2^−^ (luminal A) and triple-negative breast cancers. Within the pure luminal A (Figure 10C) and triple-negative (TNBC) subgroups (Figure 10D), no major CLDN8 differences were observed between those who responded well to chemotherapy and those who did not. In contrast, among HER2(+) patients treated with chemotherapy (often as part of combined modality regimens), CLDN8 levels were significantly higher in non-responders. Figure 10F shows that HER2^+^ tumors that failed to respond optimally had elevated CLDN8 expression, whereas responders had lower CLDN8 expression (*p* ≈ 0.01). This indicates that high CLDN8 expression may contribute to chemoresistance, particularly in HER2-driven cancers. A similar pattern emerged when focusing on hormone receptor status: in the overall ER(−) cohort, which encompassed aggressive subtypes (HER2-enriched and basal-like TNBC), non-responders exhibited higher CLDN8 expression than responders (Figure 10B, *p* = 0.032). This difference was especially pronounced in the HER2(+)ER(−) subgroup, essentially HER2-enriched tumors, where responders had significantly lower CLDN8 levels compared to resistant cases (Figure 10H, *p* = 0.015). These findings suggest that CLDN8’s association with chemotherapy outcomes is context-dependent: in more aggressive ER(−) or HER2-driven cancers, high CLDN8 expression predicts a poorer response to chemotherapy, whereas in luminal (ER^+^/HER2^−^) cancers, CLDN8 expression has little to no predictive value for chemotherapy efficacy (Figure 10E,I,J; no significant differences in luminal A or luminal B subsets). This dichotomy aligns with the notion that CLDN8-rich tumors tend to be well-differentiated and less proliferative (features of many ER^+^ luminal cancers, which inherently respond less to chemotherapy but also have better baseline prognosis), while in inherently chemosensitive groups (like ER^−^ or HER2^+^), elevated CLDN8 acts as a resistance factor.

**Figure 11:**
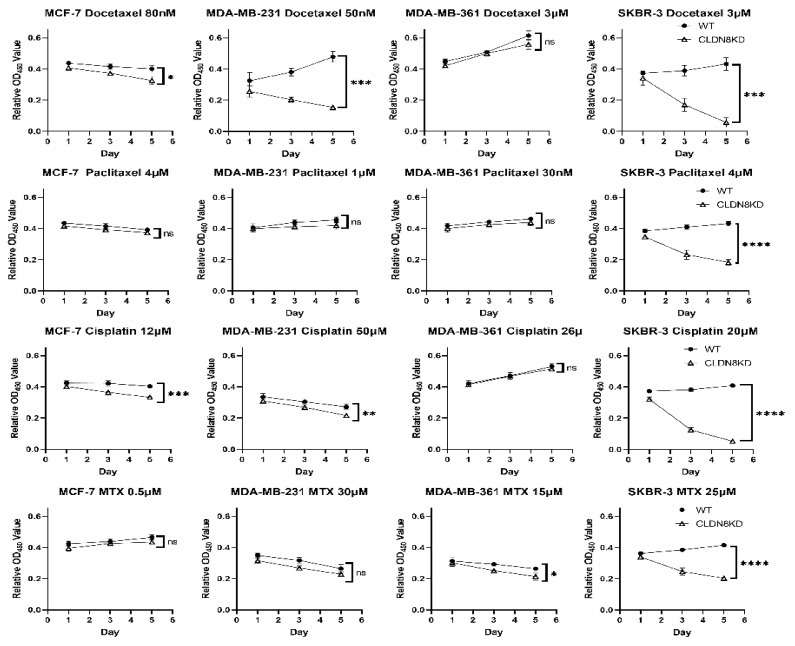
CLDN8 knockdown reduces the responsiveness to HER2-targeted therapies in breast cancer cells. Growth curve analysis of HER2(+) breast cancer cell lines treated with Lapatinib and Neratinib at sub-IC_50_ concentrations. CLDN8 knockdown significantly increased cell viability under both treatments compared to control cells, suggesting that CLDN8 enhances the efficacy of HER2-targeted therapy. Statistical significance: *p* < 0.05 (*), *p* < 0.01 (**), *p* < 0.001 (***), *p* < 0.0001 (****), relative to the corresponding WT controls.

We then performed in vitro chemosensitivity assays to validate the clinical patterns and directly test whether CLDN8 influences chemotherapy response in breast cancer cells. Figure 11 shows the growth inhibition results for representative chemotherapeutic drugs in the WT and CLDN8KD cell models. Consistent with our earlier IC50 analysis, CLDN8 knockdown enhanced sensitivity to taxanes (Docetaxel and Paclitaxel), with significant reductions in IC50 values (*p* < 0.05). However, differences in Cisplatin and Methotrexate sensitivity were less pronounced and did not reach statistical significance (*p* > 0.05), suggesting that CLDN8’s role in chemoresistance may be context-dependent and limited to specific drug classes. Figure 11 shows the effects of Docetaxel (a microtubule-stabilizing agent), Paclitaxel (a related taxane), and Cisplatin (a DNA cross-linking agent) on cell proliferation over time. Each drug was applied at a fixed concentration near the IC50 for WT cells, and the cell counts were tracked for up to 5 days. Under Docetaxel treatment (Figure 11), CLDN8KD cells showed markedly greater growth suppression than WT cells across multiple lines. By day 5, MCF-7 (luminal) and MDA-MB-231 (triple-negative) CLDN8KD cultures had 25–30% fewer viable cells than their WT counterparts treated in parallel, reflecting a significantly enhanced drug effect (*p* < 0.05 for MCF-7; *p* < 0.001 for MDA-MB-231). In SKBR-3 cells (HER2^+^), which are moderately sensitive to Docetaxel, CLDN8 knockdown led to an even more pronounced reduction in proliferation. CLDN8KD SKBR-3 cells showed virtually no net growth over 5 days, whereas WT SKBR-3 cells continued to expand, yielding a highly significant difference (** *p* < 0.001). Notably, in the MDA-MB-361 (ER^+^/HER2^+^) line, which has a relatively epithelial and slower-dividing character, Docetaxel inhibited growth similarly in WT and KD (no significant difference by day 5), consistent with the clinical finding that CLDN8 is less predictive in luminal contexts.

The Paclitaxel results (Figure 11) similarly demonstrated heightened chemosensitivity with CLDN8 silencing. All CLDN8KD lines tended toward lower cell viability under Paclitaxel than WT, but the effect was most dramatic in SKBR-3 cells: WT SKBR-3 proved partially resistant, maintaining proliferation under 10 nM Paclitaxel, whereas CLDN8KD SKBR-3 cells were nearly completely growth-arrested, leading to an approximately 50% lower cell count than WT by day 5 (*** *p* < 0.0001). This stark contrast in the HER2^+^/ER^−^ SKBR-3 mirrors the clinical observation (Figure 10H,F) that high CLDN8 expression is linked to poor chemo-response in HER2-driven cancer; when CLDN8 is removed, the cells succumb much more readily to the taxane. MCF-7 and MDA-MB-231 KD cells also showed greater growth inhibition than WT under Paclitaxel (with modest but significant differences by the end of the experiment, *p* < 0.05), whereas MDA-MB-361 again showed only a minor enhancement with CLDN8KD.

Finally, Cisplatin treatment (Figure 11) reinforced this trend. Cisplatin caused substantial cell death in all models, but CLDN8KD cells exhibited deeper and more rapid reductions in viability than WT cells. For example, in MCF-7 cells, 12 µM Cisplatin progressively reduced WT viability over 6 days, but CLDN8KD MCF-7 cells underwent an even sharper decline, resulting in a significantly lower survival fraction by day 6 (** *p* < 0.001). MDA-MB-231 (TNBC) cells also showed enhanced Cisplatin sensitivity with CLDN8 knockdown: although both WT and KD were affected by a high dose (50 µM), CLDN8KD MDA-MB-231 cells had a markedly greater loss of viability by day 5 (*p* < 0.01 vs. WT). Interestingly, MDA-MB-361 (luminal B) responded similarly to Cisplatin, regardless of the presence of CLDN8 (no significant WT–KD difference at the tested dose), paralleling the lesser clinical relevance of CLDN8 in luminal chemo-response. Together, the growth assays across these chemotherapeutics demonstrated that loss of CLDN8 consistently heightened chemo-induced growth inhibition, particularly in aggressive cell line models, whereas CLDN8-expressing cells were comparatively more drug-resistant. These experimental findings strongly support the clinical data in Figure 10, indicating that CLDN8 serves as a protective factor against chemotherapy in certain subtypes. High CLDN8 levels help tumor cells withstand cytotoxic stress, likely by preserving epithelial integrity and activating survival pathways. In contrast, knocking down CLDN8 abrogated this protection, rendering the cells more vulnerable to drug-induced death. In summary, our results indicate that CLDN8 is a context-dependent modulator of treatment response, enhancing responsiveness to endocrine therapy but conferring resistance to HER2-targeted treatments and conventional chemotherapy.

## 3. Discussion

This study systematically explored the role of CLDN8 in breast cancer progression, prognosis, and treatment responses. Immunohistochemistry (IHC) analysis revealed a significant reduction in CLDN8 expression as the tumor grade and TNM stage increased. In normal breast tissue, CLDN8 is primarily localized to the cell membrane, whereas in high-grade tumors, its expression is diffuse and cytoplasmic. This shift in localization suggests a loss of tight junction integrity, which may contribute to increased tumor aggressiveness and metastatic potential. Furthermore, CLDN8 expression varied among different molecular subtypes, with ER(+) and HER2(+) tumors exhibiting moderate expression levels, whereas TNBC displayed significantly lower or undetectable expression levels. These findings highlight the complex role of CLDN8 in maintaining epithelial structure and regulating tumor progression. Clinical data analysis demonstrated that high CLDN8 expression was associated with improved disease-free survival (DFS), particularly in ER- patients, whereas no significant association was observed with overall survival (OS). The lack of correlation with OS suggests that while CLDN8 may influence early tumor progression, other factors may contribute to long-term survival outcomes.

High CLDN8 expression appears to enhance hormone-driven tumor differentiation while buffering cells against cytotoxic stress, explaining its context-dependent effects. In one study, CLDN8 was enriched in luminal breast cancers (ER+/PR+), correlating with low grade, low Ki-67, and better prognosis [21]. This tight junction protein helps maintain epithelial polarity and cell–cell adhesion, which supports an endocrine-responsive phenotype. For example, CLDN8 knockdown in ER(+) cell models led to reduced efficacy of endocrine treatments (Tamoxifen, Fulvestrant, Anastrozole), suggesting that CLDN8 preserves estrogen receptor signaling and epithelial gene expression needed for anti-estrogen therapies to work. Consistently, patients with high CLDN8 expression have improved survival with endocrine therapy. One reason is that well-differentiated, CLDN8-rich tumors remain dependent on hormone pathways and lack mesenchymal traits, making them more susceptible to growth arrest by endocrine therapy. CLDN8’s co-expression with the androgen receptor (AR) in luminal tumors further underscores its association with a hormonally regulated, less aggressive state [20]. Notably, AR-driven CLDN8 can activate pro-growth MAPK/AKT signaling in certain contexts, but in ER(+) breast cancer, this may paradoxically reinforce luminal characteristics that endocrine therapy can target effectively [22].

This observation aligns with our in vitro findings, where CLDN8 knockdown led to increased resistance to endocrine therapies, including Tamoxifen, Fulvestrant, and Anastrozole, particularly in ER(+) models. These findings suggest that CLDN8 may play a role in maintaining ER-mediated signaling, thereby influencing the efficacy of endocrine therapy.

In contrast to its beneficial role in endocrine therapy response, high CLDN8 expression was associated with reduced sensitivity to chemotherapy. Patients with elevated CLDN8 levels demonstrated poorer responses to chemotherapeutic agents, a trend further validated through in vitro experiments. CLDN8 knockdown significantly decreased the IC50 values for Docetaxel, Paclitaxel, Cisplatin, and Methotrexate, indicating that CLDN8 may contribute to chemotherapy resistance by preserving epithelial characteristics and reducing apoptotic susceptibility.

CLDN8-rich tumors tend to be more epithelial and less proliferative, which is unfavorable for chemotherapy response [23]. Strong tight junctions and cell polarity can impede drug penetration and foster anti-apoptotic signals via cell–cell contact. Indeed, we observed that knocking down CLDN8 significantly increased sensitivity to chemotoxic agents (e.g., Docetaxel and Cisplatin), lowering the IC50 and enhancing cell death, whereas CLDN8-intact cells were relatively chemoresistant. This aligns with reports that high claudin expression can confer drug resistance; for instance, claudin-7 and claudin-1/2 upregulation promotes Cisplatin resistance in pancreatic and lung cancer cells [22]. Intact junctions may prevent chemotherapy-induced apoptosis by sustaining survival pathways and preventing complete epithelial-to-mesenchymal transition (EMT). In other cancers, CLDN8 suppresses EMT and invasiveness via the AKT pathway, meaning its loss leads to a more mesenchymal, chemosensitive state [22]. Conversely, CLDN8 retention maintains cells in an adherent, less apoptotic configuration.

Another critical finding was the inverse relationship between CLDN8 expression and anti-HER2 therapy response. Patients with lower CLDN8 levels exhibited better responses to HER2-targeting agents, and this observation was supported by in vitro experiments, where CLDN8 knockdown led to significantly lower IC50 values for Neratinib and Lapatinib. These results suggest that CLDN8 may modulate HER2 signaling pathways and contribute to resistance mechanisms in HER2(+) breast cancer.

Similarly, a tight junction–rich microenvironment hinders effective HER2-targeted therapy. Anti-HER2 monoclonal antibodies (e.g., trastuzumab) partly rely on immune-mediated mechanisms and tumor accessibility; CLDN8-high tumors, being more “solid” and less infiltrated, may respond suboptimally. Moreover, CLDN8’s association with AR and epithelial differentiation implies that CLDN8-high HER2(+) cells might activate alternate survival pathways or be less “addicted” to HER2 signaling. This could reduce their dependence on HER2 and blunt the efficacy of HER2 blockade. Supporting this, researchers have found that breaking the integrity of tight junctions can improve treatment outcomes. For example, an anti-CLDN4 antibody increased Paclitaxel uptake and apoptosis in breast cancer models, enhancing chemosensitivity and reducing metastasis [24].

Taken together, these findings suggest that CLDN8 could serve as a biomarker for predicting therapeutic response and guiding personalized treatment strategies. Its biological role shifts with the therapeutic context: it promotes a polarized, hormone-sensitive phenotype (beneficial for endocrine therapy), but that same epithelial fortitude inhibits drug penetration and apoptosis under chemotherapy or HER2-targeted attack, leading to treatment resistance. This dualistic behavior of CLDN8 exemplifies how a tight junction protein can function as a double-edged sword in cancer therapy, reinforcing the need to consider the tumor context when predicting treatment response.

Despite the promising insights gained from this study, several limitations should be acknowledged. The reliance on retrospective clinical data necessitates prospective validation in larger cohorts. Additionally, while in vitro experiments confirmed the functional impact of CLDN8 on drug sensitivity, further mechanistic studies are required to identify the specific pathways through which CLDN8 modulates these effects in vivo. Investigating potential combination therapies targeting CLDN8 alongside conventional treatments could provide novel strategies for overcoming drug resistance in patients with breast cancer.

## 4. Materials and Methods

### 4.1. Cell Lines

The cell lines used in this study were obtained from the American Type Culture Collection (ATCC) (LGC standard, Teddington, UK), and comprised four human breast cancer cell lines: MDA-MB-231, MDA-MB-361, MCF-7, and SKBR-3. MDA-MB-231 and MCF-7 cells were cultured in Dulbecco’s Modified Eagle Medium (DMEM), while MDA-MB-361 and SKBR-3 cells were cultured in RPMI-1640 Medium. The culture medium was supplemented with 10% fetal calf serum (FCS) (Sigma-Aldrich, Dorset, UK) and 1× antimicrobial solution (Sigma-Aldrich, Dorset, UK). Cells were maintained in a controlled environment with a pH level of 7.3, 95% humidity, 5% CO_2_, and a temperature of 37 °C within an incubator.

### 4.2. Drugs and Antibodies

Four chemotherapy agents—Paclitaxel, Docetaxel, Cisplatin, and Methotrexate (MTX)—along with two anti-HER2 inhibitors (Neratinib and Lapatinib) and three endocrine therapies (Tamoxifen, Fulvestrant, and Anastrozole) were obtained from Sigma-Aldrich (Dorset, UK). Drug stock solutions were prepared in DMSO, with a final solvent concentration ≤ 0.1% in all treatments, which is below the cytotoxic threshold for breast cancer cells. Vehicle controls (0.1% DMSO) were included in all experiments to confirm that the solvent exposure did not affect cell viability. For protein blotting, the following antibodies were used: mouse anti-human GAPDH (SC-32233) from Santa Cruz Biotechnologies Inc. (Santa Cruz, CA, USA), and rabbit anti-human CLDN8 (710222) from Thermo Fisher (Oxford, UK).

### 4.3. Tissue Microarray (TMA) and Immunohistochemistry (IHC)

Breast cancer tissue microarray slides (BR1503f) (https://tissuearray.com) were used in this project. Immunohistochemistry (IHC) was performed as described previously. The TMA slide was dewaxed in xylene and rehydrated in a graded series of ethanol/distilled water solutions. Heat-induced antigen retrieval was performed using citrate buffer (pH 6.0) for 20 min in a microwave. After cooling, the slides were blocked with PBS containing 5% horse serum for 2 h at RT.

The sections were then incubated overnight at 4 °C with a primary antibody against CLDN8 (1:200; Abcam, Cambridge, UK, ab 211439). After washing thoroughly with PBS, the staining protocol was performed using the Vectastain Universal Elite ABC Kit (cat no. PK-6200; Vectastain Universal Elite ABC Kit, Vector Laboratories, Inc., Newark, NJ, USA) This was followed by the manufacturer’s protocol. Briefly, using the reagent from the kit, the sections were incubated for 30 min with biotinylated secondary antibody, washed with PBS, and then incubated at room temperature for 30 min with ABC tertiary reagent. The staining was then developed using 3,3′-Diaminobenzidine (DAB) substrate for 10 min. Following a brief wash in tap water, the slide was counterstained with Gill’s haematoxylin, then washed in tap water, dehydrated in a graded series of ethanol, cleared in xylene, and finally mounted with DPX (Dibutylphthalate Polystyrene Xylene). A malignant adrenal pheochromocytoma sample, denoted as “Adr” on the tissue microarray slide, was included as a positive control for the IHC experiment. This tissue type has been previously validated to express tight junction proteins, including CLDN8, and was used to confirm the accuracy of antibody binding and consistency of the chromogenic detection process. The presence of distinct positive staining in the control adrenal tissue indicated proper assay performance and ensured the validity of subsequent sample interpretations.

Staining evaluation was performed under a 40× objective based on the percentage of CLDN8-positive cells and staining intensity in two randomly selected fields. The proportion of positive tumor cells was categorized as follows: no positive cells (0), <25% positive cells (1), 25–50% (2), 50–75% (3), and >75% (4). The staining intensity was graded as follows: unstained (0), light-brown (1), brown (2), and dark-brown (3). The staining index (SI) was calculated using the formula SI = staining intensity × proportion of positively stained cells. CLDN8 expression was evaluated using the SI-scored method, with cut-off points of ≤3 and >3. As previously reported, the staining score for CLDN8 was determined by considering the extent of tumor coverage and the proportion of positive staining.

### 4.4. Tissue Cohort

As previously documented [25], we used a freshly frozen cohort of breast cancer tissues comprising both tumor and adjacent normal mammary tissues. Transcript abundance of CLDN8 was quantified by real-time PCR (qPCR), with subsequent correlation analyses performed between expression profiles and clinical parameters, including lymph node metastasis status, distant metastasis, histological grade, and 6-year survival outcomes. Written informed consent was obtained from all patients before participation, and the study received ethical approval from the Bro Taf Health Authority (ethics approval No. 01/4303 and 01/4046). Following surgical procedures, patients were monitored in a follow-up study with a median follow-up duration of 120 months post-surgery.

### 4.5. Patient Response to Chemotherapy and Evaluation

In this study, we utilized an extensive public database containing records of patients with breast cancer along with their corresponding therapeutic interventions (ROC Plotter—Online ROC analysis (accessed on 1 December 2023). The database used receiver operating characteristic (ROC) curve analysis to classify patients based on their responsiveness to specific therapies. The area under the curve (AUC) values and statistical measures of treatment sensitivity were recorded. Additionally, the gene expression levels of the selected targets were analyzed, with statistical significance assessed using the Mann-Whitney *U* test.

### 4.6. Breast Cancer Cell Model

Four breast cancer cell lines, specifically MDA-MB-231, SKBR-3, MCF-7, and MDA-MB-361, were selected for their representation of diverse subtypes within breast cancer pathology. These cell lines were used to create sublines characterized by CLDN8 knockdown, adhering closely to the procedural guidelines provided by the manufacturer. The small interfering RNA (siRNA) designed to target human CLDN8 (SC-44865) was acquired from Santa Cruz Biotechnologies Inc. (Santa Cruz, CA, USA).

### 4.7. RNA Extraction and Reverse Transcription

Total RNA was extracted from the tissue samples and breast cancer cell lines using the TRI Reagent Kit (Merck KGaA, Darmstadt, Germany) according to the manufacturer’s protocol. After isolation, RNA concentrations were adjusted to 500 ng/μL, and reverse transcription was performed using the GoScript™ Reverse Transcription System Kit (Promega Corporation, Madison, WI, USA) in a SimpliAmp thermocycler (Fisher Scientific UK, Leicestershire, UK). The resulting cDNA was stored at −20 °C until further analysis.

### 4.8. Real-Time Quantitative PCR (qPCR)

CLDN8 transcript expression was quantified in tissue cohorts using the Amplifluor Uniprimer™ Universal qPCR system (Intergen Inc., Oxford, UK). Forward and reverse primers were designed with a Z sequence (5′-ACTGAACCTGACCGTACA-3′) to enable the incorporation of the FAM-tagged Uniprimer™ probe for fluorescent detection. The primer sequences were as follows: CLDN8 forward primer, ACTGAACCTGACCGTACAAGCTACTGCTCTTTTCGTTG, and Z-tagged reverse primer, ACTGAACCTGACCGTACAAGCTACTGCTCTTTTCGTTG. The internal standard GAPDH forward primer sequences used were 5′-CTGAGTACGTCGTGGAGTCc-3′ and the GAPDH ZR primer sequence was 5′-ACTGA ACCTGACCGTACAGAGATGATGACCCTTTTG-3′. Each qPCR reaction included forward and reverse primers, cDNA from tissue samples, Uniprimer™, and 2× Precision FAST qPCR master mix (Primer Design, Eastleigh, UK). Real-time PCR was performed using a StepOnePlus™ Real-Time PCR System (Thermo Fisher Scientific, Leicestershire, UK) under the following cycling conditions: an initial denaturation step at 95 °C for 10 min, followed by 100 cycles of 95 °C for 10 s, 55 °C for 35 s, and 72 °C for 10 s. Transcript levels were quantified relative to an internal reference gene with known transcript copy numbers. A series of standard samples, ranging from 10^8^ to 10^1^ copies, were included on the same qPCR plates as the test samples, under identical conditions. A standard curve generated from these standards was used to determine the relative transcript copy numbers in unknown samples.

### 4.9. Western Blotting (WB)

Sodium dodecyl sulfate-polyacrylamide gel electrophoresis (SDS-PAGE) and Western blotting were performed as follows. Proteins were extracted from the cultured cells using RIPA buffer and quantified using a Bio-Rad protein quantification kit (Bio-Rad Laboratories, Hertfordshire, UK). The extracted protein samples were mixed with 2× Laemmli sample buffer, heated at 100 °C for 5 min, and loaded onto a 10% SDS-PAGE gel for separation. Following electrophoresis, proteins were transferred onto a pre-activated PVDF membrane using a semi-dry transfer system, with methanol treatment applied prior. The membrane was blocked with 10% milk to prevent non-specific binding, followed by incubation with primary antibodies targeting CLDN8 (1:500; Abcam, ab 211439) and β-actin (1:5000; SANTA CRUZ, sc-47778). After primary antibody incubation, the membrane was treated with horseradish peroxidase (HRP)-conjugated secondary antibody. Protein detection was performed using the EZ-ECL chemiluminescent reagent (Geneflow Ltd., Litchfield, UK).

### 4.10. In Vitro Drug Sensitivity Test

Cells were seeded into 96-well plates and treated with a series of drug dilutions at a 1:10 ratio. The selected drug concentrations were based on the established IC50 values and previous experimental data. After a 72-h incubation, the cells were fixed with 4% formalin, stained with 0.5% crystal violet, and washed before being solubilized in 10% acetic acid. Absorbance was measured at 595 nm using a spectrophotometer to determine the cell density. Drug toxicity was calculated using the following formula: Percentage drug toxicity = [(Absorbance of untreated control − Absorbance of drug-treated sample)/Absorbance of untreated control] × 100. Scatter plots were generated to visualize the relationship between drug concentration and toxicity, and the IC50 values were determined using the best-fit curve method.

### 4.11. Thiazolyl Blue Tetrazolium Bromide (MTT) Based Cellular Growth Assay

MTT-based assays were performed to assess the impact of CLDN8 on cell proliferation. Briefly, 2 × 10^4^ cells from each cell model were plated in triplicate onto three separate 96-well plates and incubated at 37 °C with 5% CO_2_. On days 1, 3, and 5, 22 μL of 5 mg/mL MTT solution (Sigma-Aldrich Co., Poole, Dorset, UK) was added to each well, followed by a 4-h incubation at 37 °C with 5% CO_2_. After incubation, the medium was removed, and 100 μL of dimethyl sulfoxide (DMSO) (Sigma-Aldrich Co., Poole, Dorset, UK) was added to each well to dissolve the formazan crystals. The plates were then incubated for an additional 10 min at 37 °C with 5% CO_2_, and absorbance was measured at 540 nm using an LT4500 plate reader (Wolf Laboratories, York, UK).

### 4.12. Statistical Analysis

Statistical analyses were conducted using the SPSS software (Version 27.0; IBM Corp., New York, NY, USA). Group comparisons were evaluated using the Kruskal−Wallis test and analysis of variance (ANOVA), where applicable. The Mann−Whitney *U* test was used for pairwise comparisons, as detailed in the text. Survival analysis was performed using the Kaplan−Meier method with log-rank testing. Cox regression modeling was applied for both univariate and multivariate analyses. Classification assessments were performed using Receiver Operating Characteristic (ROC) analysis. A *p*-value of <0.05 was considered statistically significant.

## 5. Conclusions

This study highlights the complex and context-dependent roles of CLDN8 in breast cancer progression and treatment response. While high CLDN8 expression is associated with a favorable prognosis in endocrine therapy-treated patients, it may also contribute to resistance to chemotherapy and anti-HER2 therapy. These findings underscore the potential of CLDN8 as a predictive biomarker and therapeutic target, emphasizing the need for further research to optimize personalized treatment approaches for breast cancer management.

## Figures and Tables

**Table 1:** Statistical analysis of CLDN8 expression across breast cancer grades, TNM stages, and molecular subtypes.

Total Cases		Intensity				Statistical Significance	
Entire Cohort		0	1	2	3	Ch-Square	*p*-Value
Adjacent normal breast tissue	6	0 (0%)	1 (16.67%)	3 (50%)	2 (33.33%)		
Tumor	144	33 (22.9%)	65 (44.8%)	30 (20.8%)	10 (7%)		
Pathology type							
Invasive ductal carcinoma	116	28 (24%)	49 (42%)	29 (25%)	10 (9%)		
Intraductal carcinoma	14	5 (42%)	7 (58%)	0 (0%)	0 (0%)		
Fibroadenoma	6	0 (0%)	5 (83%)	1 (17%)	0 (0%)		
lowly malignant cystosarcoma phyllodes	4	0 (0%)	4 (100%)	0 (0%)	0 (0%)		
Grade							
Grade1	8	0 (0%)	2 (25%)	5 (62.5%)	1 (12.5%)		
Grade2	80	11 (13.8%)	48 (60%)	18 (22.5%)	5 (6.3%)	7.69	0.053 ^a^
Grade3	28	11 (39.3%)	10 (35.7%)	4 (14.3%)	1 (4%)	9.61	0.022 ^a^
T stage							
T1	6	2 (33%)	2 (33%)	2 (33%)	0 (0%)		
T2	72	6 (8.3%)	44 (61.1%)	19 (26.4%)	5 (6.9%)		
T3	26	16 (61.5%)	5 (19.2%)	4 (15.3%)	1 (3.8%)	32.48	4.15 × 10^7 b^
T4	16	0 (0%)	11 (68.8%)	4 (25%)	1 (6.2%)		
Subtypes:							
ER(+)	46	10 (21.7%)	12 (26.1%)	17 (36.7%)	7 (15.2%)	2.73	0.43
HER2(+)	29	9 (31.0%)	12 (41.4%)	7 (24.1%)	1 (3.4%)	9.02	0.03 ^c^
TN	25	6 (24%)	11 (44%)	8 (32%)	0 (0%)	11.15	0.01 ^c^
ER(+)/HER2(+)	13	2 (15.4%)	3 (23%)	6 (46.1%)	2 (15.4%)	1.64	0.65 ^c^

Note: ^a^ Compared with the Grade1 group; ^b^ Compared with the T2 group; ^c^ Compared with adjacent normal group.

**Table 2:** IC50 values of the cytotoxicity assays in the breast cancer cell model.

Cell Models		Chemotherapy				Anti-Her2+ Therapy		Hormone Therapy		
		Docetaxel	Paclitaxel	Cisplatin	MTX	Neratinib	Lapatinib	Tamoxifen	Fulvestrant	Anastrozole
SKBR-3	Control	2.77 ± 0.34 µM	3.93 ± 0.38 µM	18.60 ± 1.41	23.99 ± 2.67 µM	6.62 ± 6.43 µM	5.09 ± 2.50 µM	40.95 ± 9.72 µM	9.14 ± 1.77 µM	117.31 ± 7.33 µM
	CLDN8 KD	0.86 ± 0.17 µM	1.07 ± 0.10 µM	16.96 ± 5.75 µM	20.89 ± 2.92 µM	0.78 ± -.67 µM	2.44 ± 2.32 µM	53.54 ± 13.46 µM	12.21 ± 2.83 µM	17.65 ± 2.64 µM
MDA-MB-361	Control	2.89 ± 0.40 µM	27.78 ± 0.8.05 µM	25.55 ± 4.04 µM	12.57 ± 0.34 µM	8.42 ± 6.61 µM	1.06 ± 0.10 µM	16.89 ± 2.62 µM	0.26 ± 0.05 µM	1.89 ± 0.26 µM
	CLDN8 KD	2.15 ± 0.13 µM	17.68 ± 0.7.67 µM	24.22 ± 4.58 µM	10.73 ± 1.38 µM	4.07 ± 3.05 µM	0.58 ± 0.06 µM	23.66 ± 3.35 µM	0.61 ± 0.10 µM	2.99 ± 0.42 µM
MDA-MB-231	Control	37.22 ± 9.97 µM	0.55 ± 0.05 µM	48.60 ± 7.17 µM	25.86 ± 9.01 µM	1.57 ± 0.14 µM	5.48 ± 1.34 µM	26.01 ± 6.12 µM	4.47 ± 2.03 µM	11.80 ± 2.42 µM
	CLDN8 KD	18.34 ± 9.31 µM	0.31 ± 0.02 µM	36.82 ± 7.03 µM	15.26 ± 5.28 µM	1.04 ± 0.09 µM	4.36 ± 0.80 µM	32.25 ± 6.89 µM	4.19 ± 2.03 µM	18.13 ± 3.80 µM
MCF-7	Control	74.35 ± 06.53 µM	3.68 ± 0.51 µM	11.52 ± 1.27 µM	0.41 ± 0.054 µM	0.43 ± 0.04 µM	9.33 ± 3.00 µM	12.29 ± 1.80 µM	4.11 ± 5.7 µM	0.14 ± 0.04 µM
	CLDN8 KD	54.05 ± 5.37 µM	2.66 ± 0.68 µM	8.44 ± 0.88 µM	0.12 ± 0.04 µM	0.29 ± 0.01 µM	5.58 ± 1.26 µM	17.17 ± 2.72 µM	5.76 ± 4.83 µM	0.93 ± 0.36 µM

## Data Availability

Data are contained within the article.
